# Research on interaction and trust theory model for cockpit human-machine fusion intelligence

**DOI:** 10.3389/fnins.2024.1352736

**Published:** 2024-03-04

**Authors:** Ya Duan, Yandong Cai, Ran Peng, Hua Zhao, Yue Feng, Xiaolong You

**Affiliations:** ^1^Institute of Solid Mechanics, School of Aerospace Engineering, Tsinghua University, Beijing, China; ^2^China Flight Test Establishment, Xi’an, China

**Keywords:** human-machine fusion, dynamic operational limits, human-machine trust, physical and mental characteristics, operational capabilities

## Abstract

Based on Boyd’s “Observation Orientation-Decision-Action (OODA)” aerial combat theory and the principles of operational success, an analysis of the operational division patterns for cross-generational human-machine collaboration was conducted. The research proposed three stages in the development of aerial combat human-machine fusion intelligence: “Human-Machine Separation, Functional Coordination,” “Human-Machine Trust, Task Coordination,” and “Human-Machine Integration, Deep Fusion.” Currently, the transition from the first stage to the second stage is underway, posing challenges primarily related to the lack of effective methods guiding experimental research on human-machine fusion interaction and trust. Building upon the principles of decision neuroscience and the theory of supply and demand relationships, the study analyzed the decision-making patterns of human-machine fusion intelligence under different states. By investigating the correlations among aerial combat mission demands, dynamic operational limits of human-machine tasks, and aerial combat mission performance, a theoretical model of human-machine fusion interaction and trust was proposed. This model revealed the mechanistic coupling of human-machine interactions in aerial tasks, aiming to optimize the decision-making processes of human-machine systems to enhance mission performance. It provides methodological support for the design and application of intelligent collaborative interaction modes in aviation equipment.

## 1 Introduction

John Boyd, an American military theorist, proposed the theory of “Observation Orientation-Decision-Action (OODA)” to characterize the process of aerial combat. This theory outlines the critical principles for winning air battles: the aerial combat mission can be viewed as a continuous loop of OODA cycles, where opposing forces strive to disrupt, delay, and interrupt each other’s completion of the cycle while ensuring their own initiative in finishing the OODA loop, thereby gaining a first-mover advantage.

As the requirements for operational systems, combat styles, and mechanisms of victory evolve, the capabilities of aviation equipment undergo generational leaps. The OODA loop of air combat is currently transitioning from “Capability Maneuver for Victory 1.0” to “Information Maneuver for Victory 2.0” and “Cognitive Maneuver for Victory 3.0” (Yang, 2020). OODA 3.0 not only involves the unilateral enhancement of the capabilities of combat personnel and equipment in the aerial combat environment but also breaks down the traditional boundaries between combat personnel, combat equipment, and the combat environment. It forms a tripartite fusion of human, machine, and matter, creating a synergistic intelligent system in the mode of human-machine fusion. Human-machine fusion intelligence refers to the integration of human intelligence and machine intelligence to establish a novel intelligent system, achieving a high degree of interaction and collaboration between humans and machines.

Currently, machine intelligence has deeply integrated into next-generation aircraft equipment through various roles such as flight assistants and air combat companions. The future direction of air combat human-machine fusion intelligence lies in the comprehensive utilization of machine intelligence for the rapid and accurate processing, storage, and learning capabilities of battlefield situations, along with the human intelligence for understanding, reasoning, and judgment of the situation (Liu, 2019). This entails rational fusion of human and machine intelligence, shifting away from the traditional “human-centric” human-machine operation mode towards the development of a “human-centered” air combat human-machine intelligent collaborative operation mode.

The current research on human-machine fusion intelligence is mainly focused on the application of artificial intelligence in military confrontations. There is a lack of research on how to propose a human-machine system integration route from a human perspective. Walsdorf and Onken (2000) analyzed the efficiency of operational personnel in flight missions and fatigue management methods, and proposed a cognitive human-machine cooperation model to describe the interaction and cooperation between operators and cockpit assistant systems. Brand and Schulte (2018) studied how to design a system that can automatically adjust and assist based on the pilot’s workload and evaluated the system’s performance and effectiveness through experiments. Wang and Liu (2023) focused on battlefield command and control tasks, pointing out the characteristics of human-machine functions, and proposed a situation awareness model based on human-machine fusion, aiming to combine the advantages of human intelligence and machine intelligence of commanders to make unified judgments and decisions. Sun et al. (2020) summarized the challenges in future human-machine intelligent collaboration and proposed three stages of physical interaction, digital interaction, and intelligent interaction in human-machine interaction evolution. They discussed the problems of allocation of human-machine agility, dynamic learning and correction, situational adaptation, and proactive response patterns from a technical perspective, as well as the interpretability, trust, emotionalization, and fairness and responsibility issues in collaborative experience.

Currently, there is a lack of systematic methods and theories for future human-machine fusion intelligence in aerial combat, mainly focusing on single theoretical guiding methods for safety and efficiency in human-machine decision-making, which cannot adaptively solve the human-machine fusion interaction and trust issues between aircraft equipment. This paper focuses on the mission requirements of aerial combat, summarizes and analyzes the role forms, scientific problems, and technical paths of human-machine fusion intelligence, and studies the theoretical model of human-machine fusion interaction and trust to support the design of human-machine collaboration and compatibility in future cockpit and manned-unmanned cooperative application scenarios.

## 2 The role forms of human-machine fusion intelligence in aerial combat

The fusion between human and machine intelligence is the foundation of generating new combat capabilities in intelligent aerial combat. The future human-machine fusion intelligence in aerial combat can be divided into three stages based on the degree of fusion:

The first stage is “human-machine separation and functional coordination.” Humans are the dominant force while machines have low autonomy and can only execute limited combat tasks according to programmed instructions or human commands. Humans are in control of aerial combat, and machines serve as assistants. Communication between humans and machines is done through channels such as vision, hearing, and touch, with simple division of labor in terms of functionality. Therefore, the main feature of this stage is human-machine separation, where information exchange between humans and machines is based on programs or rules. The form of human-machine fusion is mainly reflected in functional coordination, where machines act as extensions of human perception and execution capabilities, serving as “servants” and “assistants” to humans (Zhu et al., 2021).

The second stage is “human-machine trust and task coordination.” Machines have higher autonomy, and the allocation of authority between human intelligence and machine intelligence in aerial combat is dynamically determined. Machines can autonomously complete combat tasks under human authorization or supervision. The trust relationship between humans and machines is established through smoother, more efficient, and real-time task coordination in aerial combat, significantly increasing the level of human-machine fusion. In this process, humans and machines jointly face battlefield threats and carry out collaborative aerial combat tasks. Therefore, human-machine trust is critical. Machines need to improve their ability to recognize human states and enhance their understanding of human intentions. The artificial intelligence process should be as transparent as possible, providing interpretable autonomous decisions and presenting the logic, process, and results of machine actions to combat personnel to gain their trust. Combat personnel need to adapt to machines transitioning from “servants” and “assistants” to “companions” and fully leverage the advantages of machine intelligence in different tasks and scenarios, establishing sufficient trust in machines. Based on the establishment of human-machine trust, the form of human-machine fusion is reflected in task coordination, where humans and machines become “comrades” in collaborative combat.

In the third stage, “human-machine integration and deep fusion,” the autonomy of machines is further enhanced. In aerial combat, human intelligence and machine intelligence are deeply integrated and seamlessly connected. Direct communication and control channels are established between the human brain and machines through brain-machine interfaces and neural mimetic devices. Humans are able to command and control machines or a large number of unmanned systems through “mind-controlled group control.” There is a mutual understanding and fusion of thoughts between humans and machines, and the degree of human-machine integration enters an advanced stage (Sun et al., 2021). “Mind control” not only helps combat personnel to manipulate machines more flexibly and efficiently, but also enables accurate enemy-identification, long-distance real-time command, intelligent mission planning, and efficient autonomous collaboration, becoming a winning mechanism in future aerial combat. Machines, through learning, possess the ability to handle complex situations like humans, and can autonomously plan, design, and collaborate with combat personnel to complete difficult combat tasks (Liu et al., 2021). Combat personnel have a high level of trust and understanding in the decision-making and behavioral patterns of machine intelligence. The form of human-machine integration is manifested in complementing, learning from, and promoting each other’s intelligence, achieving brain-machine integration and unity. Humans and machines become “partners” with mutual understanding and empathy.

Currently, although artificial intelligence technology continues to advance and the autonomy of machines improves, machine intelligence still has certain limitations in the context of air combat. Therefore, the integration of human-machine intelligence is still in the process of transitioning from the first stage of “human-machine separation and functional coordination” to the second stage of “mutual trust and task coordination between humans and machines.” This paper focuses on the interactive and trust issues faced during the development from the first stage to the second stage of human-machine integration, and aims to construct a theoretical model to reveal the mechanism of human-machine coupling.

## 3 Human-machine coupling process based on resource supply-demand relationship

The three elements of the human-machine-environment system in the aerial combat system mainly include operators, aircraft equipment, and operating environment. The aircraft equipment includes its platform flight system, mission system, and life support system, which constitute the microenvironment and interactive environment for the work of the flight operators. The operating environment mainly focuses on the task scenarios and meteorological environment outside the equipment-based system, as well as other external battlefield environments. These three elements form a complex system through the real-time input of tasks, and the task is transformed into workload through the interaction between humans and machines. The operating environment changes the operator’s ability performance by influencing their operating state, thereby adjusting the performance output of the human-machine system. Therefore, according to the systems theory and the theory of residual resources, the second stage of human-machine fusion intelligence process can be described as a relationship of resource demand and supply, and the efficiency of the system’s work contribution to the aerial combat system can be determined by the compatibility between the two.

**Figure 1:**
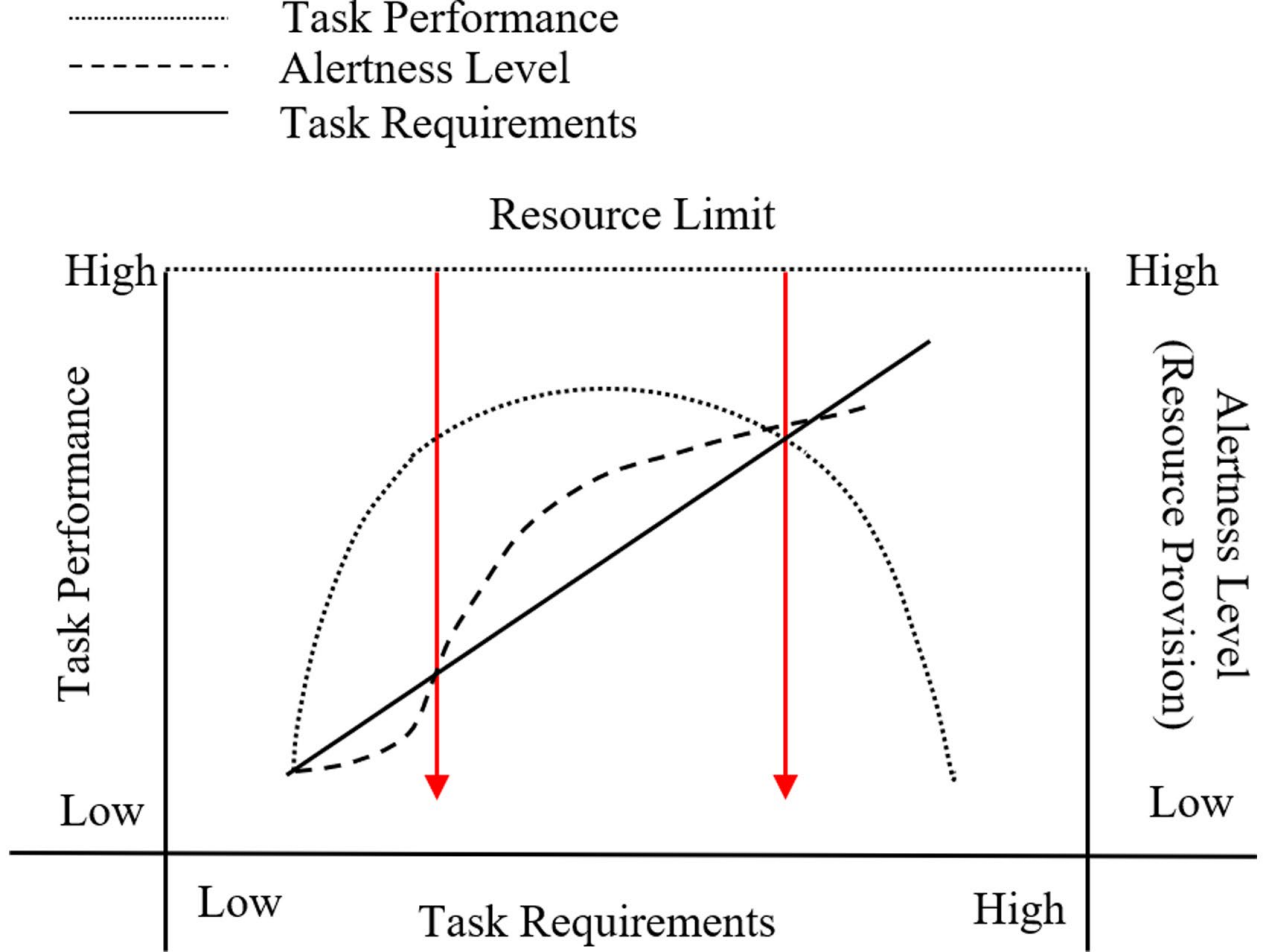
Supply and demand between workload and task performance.

As the priority “decision-maker” in the current human-machine system operation, the capability of the operators determines the boundary of the system’s effectiveness. Quantifying the operator’s capability is an important approach to achieving human-machine fusion intelligence. The residual resource theory uses the “cognitive resource” residual amount to represent the operator’s capability under certain task demand conditions, as shown in Figure 1 (Mark et al., 2015). The task demand is linearly and positively correlated with the workload, indicating that the greater the task demand, the greater the workload. However, the task demand and task performance show an inverted U-shaped relationship. Under low and high task demand conditions, the task performance shows the same declining state. Within a certain range of task stimuli, there exists an optimal performance range. The main reason affecting performance representation is the operator’s level of arousal after task stimulation. When overloaded tasks occur, the operator’s utilization of cognitive resources exceeds the effective utilization range of the maximum residual capacity, attention resources become limited, information reception channels become narrower, and the situation of mismatch between resource demand and supply arises, resulting in a decline in performance. Conversely, under low workload conditions, the level of stimulation is insufficient to arouse the operator’s interest, and the resources cannot be effectively mobilized, leading to poor task completion performance. For the aerial combat human-machine-environment system, “task demand” will no longer be limited to the operational and time resource demands of the task itself, but will also incorporate task scenarios and environment to represent the degree of external input stimulation. For example, for the same beyond-visual-range air combat task, when performed in different lighting environments at night and during the day, the workload of operators will significantly increase at night, and the resource supply required for operation will also increase.

In any resource-limited system, the most relevant measure of demand is specified relative to the available supply of resources. When demand exceeds supply, an increase in demand will result in performance degradation. The maximum resource supply capacity of workers under different task demands is defined as “dynamic tolerance,” and the cognitive resources of workers mobilized by task demands are defined as the “resource requirements” of the human-machine system. The difference between the “maximum resource supply” and the “resource requirement” is the “remaining resources.” When the “remaining resources” approach zero, job performance will be at its lowest. In engineering psychology, the “80% of maximum resource supply” of workers is defined as the peak of the “dynamic tolerance” of workers, where task performance is optimal under the stimulation of task demands. The characteristics of the dynamic tolerance are influenced by changes in the physical and mental characteristics of workers (the arousal level in Figure 1) (Zhang et al., 2021), which in turn are affected by multidimensional demands such as task operations, time, scenes, and environments. To determine the dynamic tolerance, first adjust the job environment to induce workers to be in different states of physical and mental characteristics, and then adjust the “resource requirements” until job performance begins to decline. In this state, the “resource requirements” are equal to the “dynamic tolerance,” which is used in this study to represent the boundary of a person’s job ability.

## 4 Model construction for fusion theory

### 4.1 Overall architecture

**Figure 2:**
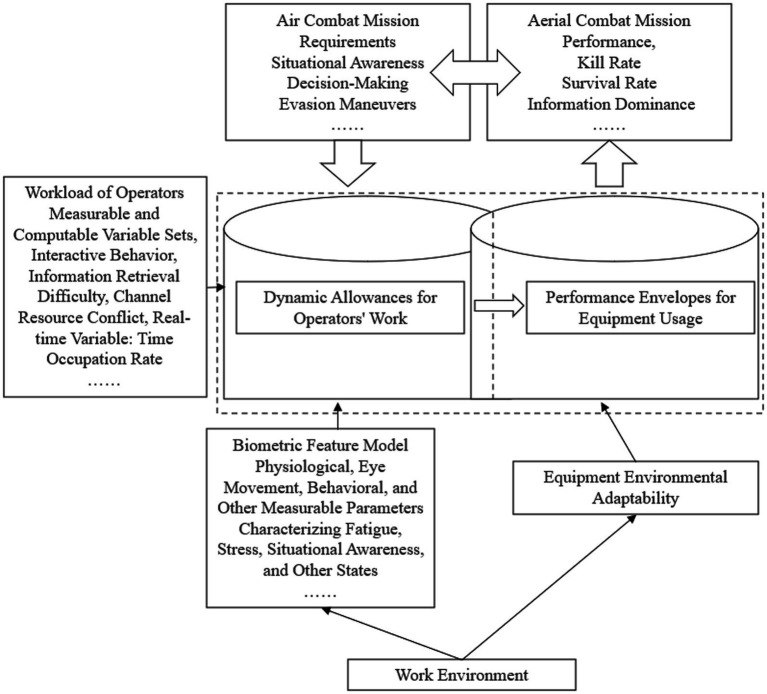
Schematic diagram of human-computer fusion interaction and mutual trust model.

In the field of aerial combat, the degree of fusion among human, machine, and environment determines the combat effectiveness. As mentioned in section 2, if the three elements of human-machine-environment are taken as inputs and transformed into the workload of operators through human-machine interaction based on the requirements of aerial combat missions, the operational environment, as a variable influencing the operational state of human-machine, interacts with the human-machine system to form a system. After undergoing functional processing, the system outputs the performance of aerial combat missions. The specific model schematic is shown in Figure 2, the effectiveness of human-machine fusion depends on the intersection of the operational capabilities of humans and machines. Under the condition where the intelligent agent has not yet formed a fully adaptive autonomous capability, the operational capability of the machine depends on the performance envelope of the designed equipment usage, while the operational capability of humans depends on their dynamic tolerance limit. Both are subject to the dynamic changes influenced by the operational environment and require real-time adjustment to match the task requirements. In the current form of equipment application, the capability boundary of the machine can be measured and adjusted, while the capability boundary of humans has not been effectively quantified. This paper focuses on studying the characteristics of human operational capability and the characteristics of the human-machine mixed operation interval in the fusion model, including:

- The differences in task requirements under different scenarios determine the characteristics of the system’s output performance in terms of individual task performance and system contribution performance.
- In the overlapping area between operators and equipment work, if there are unidentified or misidentified compensations due to unclear boundary capabilities between humans and machines, it indicates the occurrence of excessive trust or distrust issues.
- In different scenario environments, flight operators are in different states of physical and mental characteristics. These different states of physical and mental characteristics determine the flight operators’ dynamic work tolerance to varying degrees, which is referred to as the operational capability boundary.

The essence of the theoretical model for human-machine fusion intelligence interaction and mutual trust is to unveil the interconnected relationship between task requirements, psychophysiological characteristics, operational capabilities, and task performance. This model aims to provide a methodological approach for regulating task performance across three dimensions: human, machine, and environment. Upon decomposing these four main characteristics, it becomes clear that they are interlinked in a sequential relationship. The article explicates the correlation between task requirements and psychophysiological characteristics through the lens of a resource supply-demand relationship. It primarily investigates the impact of psychophysiological characteristics on operational capabilities, the link between operational capabilities and task performance, and the role these four characteristics play within the context of human-machine fusion interaction and mutual trust.

### 4.2 Intersection characteristics of human-machine operational capacity range

Based on the performance envelope of equipment development and testing, the working range of the machine can be determined. Through the model of physical and mental characteristics and dynamic capacity boundaries, the operational range of personnel can also be derived. There will inevitably be a dynamically changing overlap region between the two. On one hand, changes in the battlefield environment can lead to changes in the physical and mental characteristics of operators and performance degradation of equipment, thereby affecting the changes in the overlap region. On the other hand, as the level of machine intelligence improves and the training and capabilities of operators improve, the overlap region will become larger.

**Figure 3:**
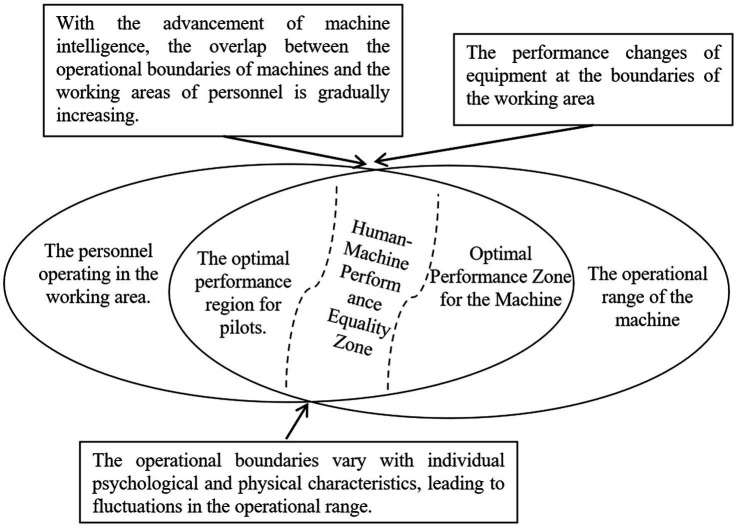
Top view of the human-machine fusion interaction and mutual trust theoretical model.

When the operational task demands fall within non-overlapping areas, there is a clear allocation of responsibilities between humans and machines, allowing the human-machine system to perform with expected and stable capabilities. However, when the operational task demands fall within the overlapping area, conflicts arise regarding who should take on the demands, leading to difficulties in establishing a definitive boundary for the allocation of responsibilities between humans and machines. The boundaries of the human-machine operational range are dynamically influenced by environmental changes, further complicating the determination of the specific division of responsibilities (see Figure 3).

Regarding the capabilities curve of machines within the full performance envelope in typical scenarios and the operational effectiveness, research and exploration in the field of weapon and equipment testing and evaluation have formed a basic model-based dynamic representation (Zhou et al., 2020). Operators can dynamically grasp the boundary area of machine operational capabilities through learning, training, or real-time prompts on the machine. However, there is currently no model-based representation of the capabilities curve for operators. If flight operators exhibit subjective over-reliance, distrust, or objective changes in the environmental impact on operator capability boundaries, there may be phenomena such as mismatched permissions and conflicts.

To resolve such conflicts, it is necessary to achieve standardized descriptions of capabilities and performance for both humans and machines in different scenarios, as well as in boundary and overlapping areas. This can be achieved through model analysis based on the fusion of interaction and mutual trust states, in order to seek the optimal solution for dynamic matching between humans and machines in overlapping areas.

### 4.3 Differences in task performance and air combat performance characteristics

The task requirements of air combat are achieved through scene interaction between humans and machines, which matches the interactive resources in the cockpit and the target resources in the scene. Therefore, the performance of the human-machine environment system is mainly manifested in task performance and air combat performance. Task performance refers to the effectiveness and efficiency of completing specific tasks, generally including the degree of task completion, the time and resources spent, and the quality of task execution. It is usually used to measure the performance and results of a team or individual during task execution, and is an important indicator for evaluating task execution capabilities and efficiency. For example, in task scenarios with clear operational goals such as shipborne training and aerial refueling, task performance represents the output effectiveness of the human-machine environment system. Air combat performance refers to the performance and results in aerial combat, generally including maneuverability, survivability, rapid targeting ability, combat firepower, situational awareness ability, maneuvering ability, electronic countermeasures ability, combat radius, etc. It is usually used to evaluate the combat capabilities of pilots and aircraft and the contribution of the system, and is of great significance in determining the outcome of aerial combat. For example, in beyond visual range air combat and close combat scenarios, feature indicators based on target requirements are selected to represent different air combat effects. In addition, both task performance and air combat performance are characteristics related to the relationship between task resource demand and supply. From the perspective of interaction between humans and machines, there is operational interaction efficiency between the two, which is generally used to measure the correlation between resource occupation and resource supply on different interaction channels of humans. It is usually used to support the rational evaluation and design optimization of human-machine interaction methods.

Task performance focuses on the ability and effectiveness of pilots in completing tasks, while air combat performance focuses on the combat ability and effectiveness of pilots in aerial combat, and there is a progressive relationship between the two. In aerial combat, excellent operational interaction efficiency between humans and machines is a prerequisite for task performance, and pilots need to comprehensively use flying skills, navigation abilities, and combat skills to complete tasks. The quality of task performance directly affects the performance of pilots in air combat. At the same time, the improvement of combat skills and combat experience can have a reverse effect on the abilities and effectiveness of pilots in task execution. Therefore, when evaluating the effectiveness of human-machine fusion, it is necessary to clarify the differences in task performance goals and select representative indicators accordingly.

### 4.4 Correlation between physical and mental characteristics and dynamic capacity limit

Dynamic capacity limit refers to the performance of individuals in different task scenarios, which is influenced by the demand for performance and the core capabilities required for the task. From the perspective of the OODA loop in aerial combat, the human-machine system needs to possess comprehensive abilities in observation, judgment, decision-making, and execution. The machine’s capability limit can be determined relatively accurately through simulation calculations and flight corrections. On the other hand, the human’s capability limit can dynamically change based on the physical and mental state of the operator and the task scenario. The performance of cognitive tasks is determined by the individual’s physical and mental characteristics. When operating in the air, the operator’s physical and mental characteristics mainly manifest as physiological factors such as hypoxia and loss of consciousness due to overload, as well as psychological factors such as overload, fatigue, and stress (Xue and Wang, 2021). The specific mapping relationship with capabilities is as follows:

- The ability of “observation, judgment, and decision-making” can be represented by the operator’s situational awareness state. Situational awareness can be divided into three relative levels: perception, understanding, and prediction (Deng et al., 2020). It mainly refers to the perception of elements in the environment, understanding their significance, and predicting their subsequent states within specific space and time. Situational awareness is a core element that affects decision-making quality and task performance. The complex and ever-changing battlefield often requires operators to maintain a high level of situational awareness. Statistics from aviation accident research reports indicate that 35.1% of non-serious accidents and 51.6% of serious accidents are caused by decision-making failures of flight operators. The main reason for decision-making failure is not due to errors in the decision-making process itself, but rather due to incorrect or lack of situational awareness.
- Stress is an emotional state caused by dangerous or unexpected changes in external circumstances and is a psychological factor that exists in decision-making activities (Xu and Ge, 2020). During aerial combat missions, flight personnel face high levels of pressure and tension. Moderate stress can stimulate the potential and courage of flight operators, enabling them to demonstrate higher reaction speed and better decision-making abilities. However, it can also lead to decreased concentration, slower reaction times, and even abnormal psychological states such as anxiety and depression, which can affect information perception, decision-making, and execution abilities, thus reducing real-time dynamic capacity limit and resulting in decreased task performance or safety accidents, such as carrier landing scenarios.
- Flight operations personnel are often exposed to high-speed, high maneuverability, high noise, high vibration, information overload, and long flight hours, which can easily induce cognitive fatigue. Cognitive fatigue is a state of exhaustion and depletion of cognitive abilities after prolonged engagement in tasks or activities with high cognitive load. It is caused by prolonged periods of intense concentration, attention, and thinking in the brain (Onken and Walsdorf, 2001). In a state of cognitive fatigue, individuals experience slower reaction times, lack of focus, decreased decision-making abilities, and impaired working memory and information processing. It is different from physical fatigue and directly affects an individual’s thinking and cognitive abilities.
- Flight tasks often involve a large number of high-g maneuvers, which can lead to hypoxia symptoms when there is a mismatch between the aircraft’s oxygen supply capability and the individual’s oxygen demand. Hypoxia refers to insufficient oxygen supply to the body tissues, resulting in decreased oxygen content in the blood. Flight operations personnel may experience lack of concentration, delayed reactions, and decreased judgment, which can severely affect their perception of the flight environment and corresponding decision-making abilities. They may also experience symptoms such as dizziness, vertigo, and blurred vision, further impairing their operational abilities and flight safety. Research statistics show that hypoxia accounts for 21.3% to 30.2% of in-flight incapacitation events among flight crew members (Pitchammal and Sadda, 2013; Brand and Schulte, 2018).

Therefore, studying the correlation between various physical and mental characteristics of researchers and their different operational abilities is crucial to support the cognitive state and task adaptation of flight operations personnel. It is essential for improving the quality of task decision-making and task performance.

## 5 Application of the model

The theoretical model of human-machine fusion intelligence interaction and mutual trust aims to enhance the mission performance in aerial combat. It analyzes the core factors influencing intelligent interaction and trust, illustrating two pathways for optimizing mission performance from the three dimensions of human, machine, and environment: adjusting workload and operational environment.

Adjusting workload can be categorized into two modes: directly modifying task demands or adjusting the allocation of human-machine permissions. Overall task demands can be optimized based on the results of workload prediction in task design, assessing whether the performance of the human-machine system operates within an optimal range. The allocation of human-machine permissions can be adjusted through the current adaptability of human-machine interaction and the state of mutual trust to achieve optimal effectiveness for both parties. For instance, dynamic task planning can be executed through combat command, ultimately achieving dynamic matching and integration of human-machine operational capabilities in aerial combat missions.

Adjusting the operational environment primarily involves adapting the operational capabilities of individuals in real-time tasks through the adaptive optimization of the coupling relationship between the environment and human states, thereby altering task performance. For example, adjusting physical cabin environments such as sound, light, and temperature to mitigate fatigue and stress among flight personnel, or regulating stress and situational awareness states among flight personnel through interactive reminders and information support within the information environment.

The current design of immersive cabins and the development of human-machine hybrid intelligence both validate the rationality and effectiveness of the theoretical model of human-machine fusion intelligence interaction and mutual trust.

## 6 Conclusion

The article starts by analyzing the development process of operational division patterns for cross-generational human-machine collaborative tasks, based on the requirements of aerial combat mission performance. The study proposes three stages of development for the integration of human-machine intelligence in aerial combat, with a focus on the technological pathways of human-machine fusion interaction and mutual trust during the current developmental stage. Targeting the three elements in the aerial combat human-machine system, the article introduces the theoretical model of human-machine fusion interaction and mutual trust.

The model defines task demands as input and aerial combat mission performance as output, considering the influencing variable between input and output as the dynamic operational capacity limits of human and machine. The efficacy of human-machine fusion is determined by the intersection of dynamic operational limits between human and machine, based on the relationship between resource demand and supply.

By conducting a feature analysis of three key variables in the model: the intersection of human-machine operational capability ranges, model output performance, and factors influencing human operational capabilities, the article establishes an indicator system for the study of human-machine fusion interaction and mutual trust. This approach is not only applicable to current equipment with fixed human-machine interaction and task assignment methods but can also systematically support the design and capability optimization of future cockpit collaboration and manned-unmanned teaming scenarios.

## Data availability statement

The original contributions presented in the study are included in the article/supplementary material, further inquiries can be directed to the corresponding authors.

## Author contributions

YD: Conceptualization, Methodology, Writing – review & editing. YC: Conceptualization, Methodology, Writing – review & editing. RP: Conceptualization, Formal analysis, Methodology, Writing – original draft. HZ: Validation, Writing – review & editing. YF: Validation, Writing – review & editing. XY: Visualization, Writing – review & editing.
